# Gender Dysphoria and Mental Health

**DOI:** 10.1192/j.eurpsy.2022.2217

**Published:** 2022-09-01

**Authors:** M. Barbosa Pinto, F. Tavares, M. Viseu

**Affiliations:** University Hospital Center of Algarve, Portugal, Department Of Psychiatry And Mental Health – Faro, Faro, Portugal

**Keywords:** mental health, Gender Dysphoria, psychiatric disorders

## Abstract

**Introduction:**

Gender dysphoria is characterized by a mismatch between the biological sex and gender identity of a person, frequently associated to distress or discomfort. Many transgender people will seek professional help to obtain a congruence between the gender identity and the body.

**Objectives:**

Brief review of the literature in the field of mental health and gender dysphoria.

**Methods:**

Review of the literature, through research in the *PubMed* database, using the following keywords: “gender dysphoria”, “mental health”, “psychiatric disorders”.

**Results:**

Although the true prevalence of gender dysphoria (GD) is unknown, several studies indicated that the prevalence of psychiatric disorders in this population is elevated. In comparison with the general population, persons with GD have higher rates of depressive symptoms (64.5%), suicidality (42.9%), substance use disorders (40.2%), general distress (33.8%), anxiety (25.9%), discrimination, and stigma, that contribute to mental health problems. Even though, we cannot reach firm conclusions due to the lack of controlled studies exploring psychiatric disorders on GD people versus controls. An interdisciplinary approach to the health and well-being of this population is highly recommended. Social support, community connectedness, and effective coping strategies appear beneficial.

**Conclusions:**

Individuals with GD have higher rates of psychiatric disorders and social stressors. Healthcare professionals should have a basic understanding on GD. Management should be individualized and may involve a multidisciplinary team. It would be important to have access to more controlled studies in order to achieve a better characterization of the prevalence of mental health disorders in this population.

**Disclosure:**

No significant relationships.

